# From signal analysis to clinical practice: the use of peak frequency in mapping and ablation

**DOI:** 10.3389/fcvm.2026.1787169

**Published:** 2026-04-10

**Authors:** Omnia Kamel, Mohamed Abdelazem, Sherien Awad, Mohamed Sharief, Ahmed Ammar

**Affiliations:** 1 Department of Cardiology, Aswan Heart Center, Aswan, Egypt; 2 Department of Cardiology, UMass Chan-Baystate Medical Center, Springfield, MA, United States; 3 Department of Cardiology, Nasser Institute Hospital for Research and Treatment, Cairo, Egypt; 4 Cardiology Department, Lancashire Teaching Hospital NHS Foundation Trust, Preston, United Kingdom; 5 Department of Cardiology, Worcestershire Acute Hospitals NHS Trust, Worcester, United Kingdom; 6 Department of Cardiology, Ain Shams University, Cairo, Egypt

**Keywords:** peak frequency, near-field, far-field, omnipolar technology, atrial fibrillation, atrial tachycardia, ventricular tachycardia, critical isthmus

## Abstract

Differentiating near-field (NF) from far-field (FF) electrograms (EGMs) is essential for accurate mapping and ablation of cardiac arrhythmias. With the advent of high density mapping systems, this distinction has traditionally relied on bipolar voltage analysis and activation mapping, while the evaluation of signal frequency has remained largely underexplored. Recently, a novel algorithm called peak frequency (PF) has been introduced as a complementary tool to conventional mapping strategies. By applying Wavelet transformation (WT), PF enables objective quantification of signal frequency, accurate identification of FF electrograms, and visualization of high frequency components on electro-anatomical maps. This review examines the role of peak frequency in mapping and ablation, spanning from fundamental signal analysis to clinical applications.

## Graphical Abstract

**Figure:**
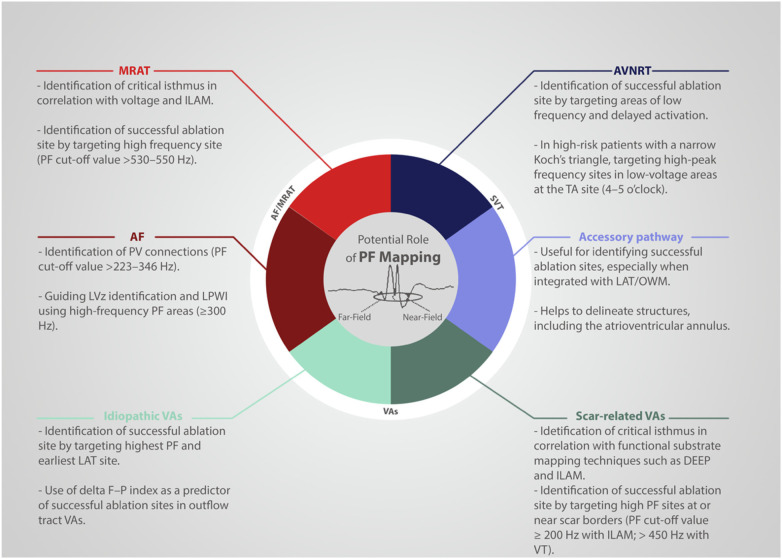
Graphical abstract summarising the potential role of the peak frequency mapping

## 1 Introduction

Intracardiac bipolar electrogram (EGM) recordings commonly contain a mixture of near-field (NF) and far-field (FF) components. Accurate differentiation between these signals is fundamental for reliable annotation of local activation time (LAT), construction of activation maps, and identification of ablation targets. Misinterpretation of FF signals as local activation may lead to erroneous mapping, whereas dismissing small-amplitude signals as FF may obscure viable myocardial tissue and critical conduction channels within scar (1).

Conventional approaches for distinguishing NF from FF activity rely primarily on bipolar voltage amplitude, EGM, timing of deflections, and slope-based measures such as dV/dt. Although clinically useful, these parameters may be limited in complex or fractionated EGMs, where amplitude and morphology do not consistently reflect the true spatial origin of activation.

Signal frequency analysis provides an additional dimension for EGM characterization by quantifying spectral content in relation to its temporal evolution (1, 2). Peak frequency (PF) is a recently introduced mapping metric that represents the highest detected frequency component within an EGM over time and is derived using Wavelet transformation (WT), which enables simultaneous temporal and frequency-domain analysis of the signal (2, 3).

This emerging technology has recently been applied to guide the mapping and ablation of various types of cardiac arrhythmias, contributing to shorter procedures, more precise identification of optimal ablation targets, and arrhythmia termination with fewer lesions, thereby improving both efficacy and safety (2, 4, 5). In this review, we critically evaluate the concept of PF-based mapping, its current clinical applications, variability in reported PF cutoff values, limitations, and potential future directions.

### 1.1 Concept

NF EGMs represent electrical activity originating from myocardial tissue in close proximity to the recording electrode. Typically, they are sharp, high in frequency, reflecting true local depolarization timing. On the contrary, FF signals record electrical activity from distant myocardium; typically, they are broader, lower in frequency, and do not reflect the actual local activation time (2).

The EnSite OT Near Field™ (OTNF) detection algorithm is a recently developed feature integrated into the EnSite × EP System (Abbott laboratories) automatically identifies NF EGMs by analysing high-frequency components using WT rather than energy-based spectral analysis. WT captures both frequency content and its temporal localization within the signal, enabling identification of localized high-frequency components in complex or low-amplitude EGMs. This approach differs from conventional time/voltage-domain features (e.g., deflection timing or slope such as dV/dt) and from Fourier-based spectral measures (e.g., dominant frequency), which summarize frequency content over a defined window and may be influenced by signal stationarity, window selection, and high-energy components. The wavelet-based approach tracks the peak instantaneous frequency over time, allowing both a PF magnitude and its timing within the EGM to be defined for each point on a map. This methodology has been implemented in automated mapping systems and validated in large cohorts of EGMs, where it was shown to highlight functionally relevant high-frequency signals during both atrial and ventricular substrate mapping (3, 6, 7).

Within contemporary electroanatomical mapping systems, including the EnSite × EP System (Abbott Laboratories), PF can be displayed on dedicated peak frequency maps or incorporated into emphasis maps, where regions exceeding operator-defined PF thresholds are highlighted within voltage or activation maps instead of relying on the earliest, latest deflection or the steepest slope (3, 6).

Importantly, PF derived from WT is independent of signal amplitude. Although PF may be displayed within systems that incorporate omnipolar signal acquisition, its calculation does not require omnipolar technology (2). Furthermore, PF analysis is not inherently vendor-dependent, as wavelet-based frequency analysis can be applied to any high-resolution EGM dataset. Offline PF analysis using exported signals from different mapping systems has also been reported; however, variability in sampling rates, filtering, and electrode configuration may influence PF estimation and limit direct cross-platform comparison (6, 7).

Reported PF cutoff values vary widely across studies, reflecting differences in chamber, rhythm, mapping density, substrate characteristics, and pharmacological state. Higher PF thresholds have been associated with critical isthmuses and slow conduction zones, but optimal values differ between atrial and ventricular arrhythmias and between sinus rhythm and tachycardia. Consequently, PF thresholds should be interpreted within the specific clinical and methodological context rather than applied as universal cutoffs (3, 7, 8).

Targeting the arrhythmogenic substrate in the scarred tissue is a well-established strategy in treating atrial and ventricular arrhythmias, especially in haemodynamic instability and non-inducibility (9). By identifying regions of high PF within low-voltage areas, PF mapping may help unmask surviving myocardial bundles embedded within scar tissue that may represent slow conduction zones or critical isthmuses involved in re-entry circuits (1).

**Figure 1:**
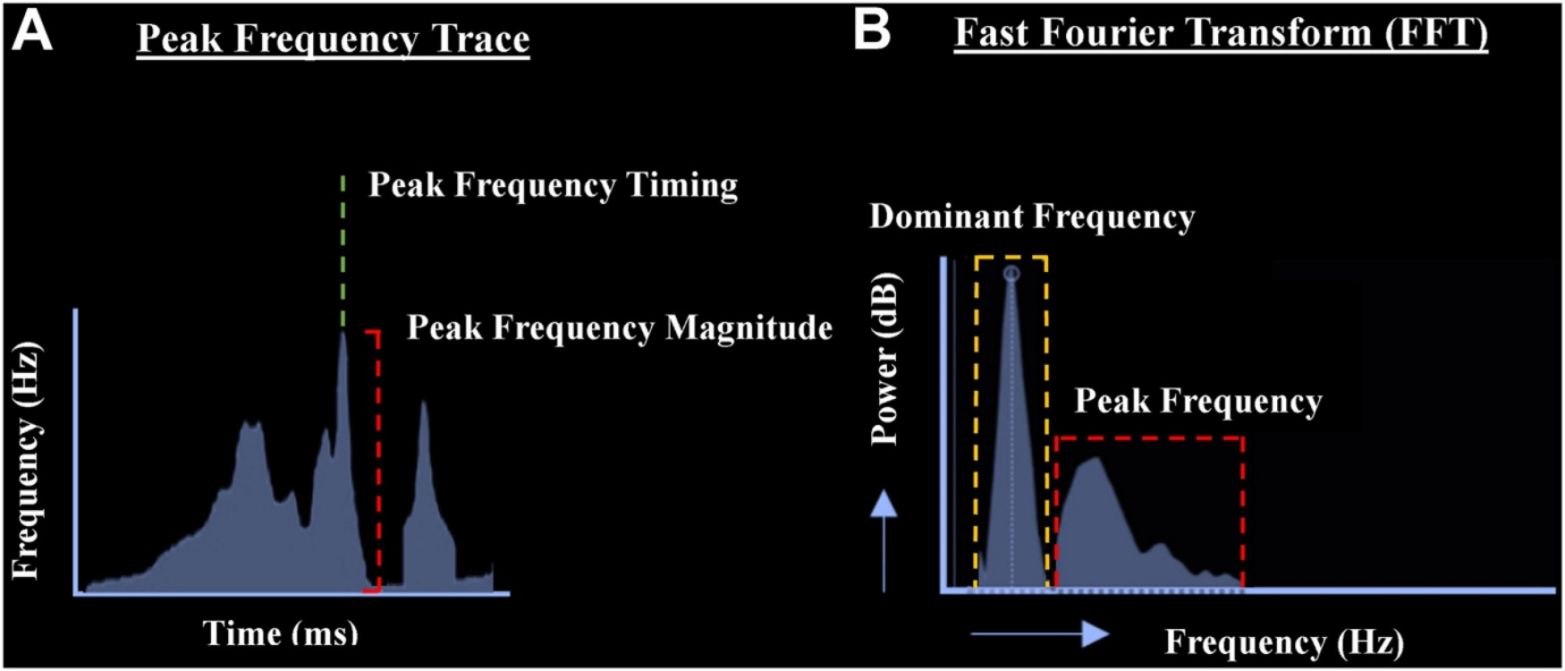
Methodology used for PF analysis and the difference to DF. **(A)** PF trace that reflects a measure of the highest signal frequencies detected in the intracardiac EGMs as a function of time. From the PF trace, 2 measures are obtained and assigned to each EGM point (1): PF timing (green line), which is derived from the time of the highest detected frequency peak. (2) PF magnitude (red line), which represents the highest frequency detected in the EGM. **(B)** Difference between PF and DF. It shows an EGM and its corresponding fast Fourier transform. The DF range (dashed orange box) represents the frequency of highest energy (typically low frequency), whereas the PF range (dashed red box) represents the highest frequencies in the EGM, often of low energy. Reproduced from [S. Al-Aidarous et al.], under the terms of the Creative Commons Attribution–Non-Commercial License (6).

Figure 1 highlights the methodology used for PF analysis and the difference from DF (6).

## 2 Role of peak frequency mapping in different types of arrhythmias

### 2.1 Atrial arrhythmias

#### 2.1.1 Atrial fibrillation (AF)

##### 2.1.1.1 Assessment of pulmonary vein isolation (PVI)

Durable PVI remains the cornerstone of AF ablation. Recognizing NF from FF electrical signals is critical in AF ablation, and PVI confirmation often relies on interpreting the “sharpness” of pulmonary vein (PV) EGMs to distinguish signals as FF or NF. This assessment is qualitative and can be challenging when FF atrial signals obscure the PV signal.

PF, as a quantitative descriptor of EGM frequency content, may assist in distinguishing NF from FF EGMs, allowing objective confirmation of PVI by differentiating PV reconnection from durable isolation, thereby providing an additional adjunctive tool to AF ablation strategies.

There have been few studies published over the last few years examining the role of PF in mapping and ablation of AF.

Kuo and his colleagues emphasized in their study the advantages of PF mapping over voltage mapping in detecting residual PV conduction gaps following PVI (10). They reported that PF criteria had higher sensitivity and positive predictive value compared to traditional voltage criteria and PF cutoff values for PV gaps were determined at 223 Hz. Comparable PF cutoff values have also been reported by Merino et al. in a Europace conference abstract, in which PF analysis was validated against bipolar voltage mapping for detection of residual PV conduction, demonstrating an optimal PF threshold of approximately 240 Hz based on receiver operating characteristic analysis (11).

Takamiya and colleagues also established typical PF values for right atrial (RA) and left atrial (LA) tissues, PVs, and superior vena cava (SVC). They found PF to be slightly higher at baseline in PVs (median 346 Hz) than LA body PFs (326 Hz). Although a relatively small difference, this may accentuate the utility of PF to distinguish NF from FF signals within PVs (12).

Furthermore, Okajima and colleagues found that PF emphasis maps improved the identification of PVI gaps and decreased the number of ablation sites needed to eliminate the gap. They found that the optimal PF cutoff for emphasis maps ranged from 315 to 437 Hz and that in 63% of the patients the re-isolation of the reconnected PV was achieved with 1 ablation application guided by PF mapping (13).

Similarly, Ting and his colleagues found that PF cutoff of 300 Hz can distinguish between FF and NF signals in PV electrograms of patients undergoing AF ablation supporting the hypothesis that PF is correlated to signal distance and that lower PF measurements are found in FF EGMs (14).

The variations in LA and PF cutoff values across studies highlight individual differences as well as the influence of the anatomic distances between the PVs, left atrial appendage (LAA), SVC, and other surrounding structures. Nevertheless, the emphasis map guided by PF annotation still provides objective identification of reconnection sites and enhances visual recognition by detecting areas of high frequency around reconnections. This approach may help reduce unnecessary ablation applications and, in turn, lower the risk of complications (13).

##### 2.1.1.2 Identification of low-voltage zones (LVZs)

In patients with persistent AF, where the efficacy of ablation remains modest despite significant advances in mapping and ablation techniques, increasing attention has been directed toward targeting additional drivers of AF beyond a PVI-only strategy (15, 16).

Early experience with substrate modification identifying and ablating proarrhythmic substrate has been encouraging. LVZs, which correlate with atrial fibrosis and scarring have been found to contribute to the arrhythmogenic substrate and independently predict AF recurrence; therefore, ablating these regions improves freedom from arrhythmia (16).

On the other hand, most studies have focused on scar homogenisation mapped at baseline sinus rhythm, leaving the role of functionally remodelled sites underexplored. Because bipolar peak-to-peak voltage mapping depends on a wavefront perpendicular to the dipole and atrial activation during AF is disorganised, LVZs may be overestimated (6).

PF mapping provides complementary information to voltage mapping by identifying regions with higher EGM frequency content and signal complexity. This approach may help to differentiate fixed from functional remodelling and may reduce the identification of spurious low-voltage zones, thereby potentially improving substrate characterization. In the study by Al-Aidarous et al., PF mapping in persistent AF patients distinguished non-LVZs, fixed remodelling, and functional remodelling sites. Specific PF thresholds were predictive: ≤214 Hz for fixed remodelling, ≥244 Hz for non-LVZs, and 215–236 Hz for functional remodelling (6).

##### 2.1.1.3 Left atrial posterior wall isolation (LAPWI)

Complete left atrial posterior wall isolation (LAPWI) is still challenging owing to overlapping epicardial conduction and risk of atrio-oesophageal fistula (17). Incorporating PF mapping to guide radiofrequency catheter ablation may help determine the optimal ablation line, thereby increasing the likelihood of achieving LAPW isolation with fewer applications (18). Furthermore, it can facilitate the distinction between epicardial and endocardial potentials, enabling precise localization of endocardial gaps along LAPWI lines (19).

Yamagami et al. used PF to guide LAPWI in patients with persistent AF by targeting ablation sites with high PF areas ablation sites (300 Hz or higher) suggesting that with good catheter contact, high PF likely marks thinner atrial myocardium or fewer epicardial bundles, where transmural conduction block lines might be easier to achieve. This promising strategy can improve the success rate of LA roof line creation and LAPWI while reducing extra radiofrequency applications and overall procedure time (18). In addition, Nishiuchi et al. also highlighted in their 2 case series that the PF value of the local potential recorded by the PFA catheter appeared to be related to the lesion formation and that the pre-pulsed field application peak frequency map may facilitate appropriate positioning of pulsed field catheters and hence allow better efficiency of PFA applications (20).

#### 2.1.2 Macro-re-entrant atrial tachycardias (MRATs)

MRATs involve circuits with slow conduction which is a key element in the mechanism of re-entrant arrhythmias. Identifying the tachycardia circuit is essential in catheter ablation strategies. However, the tachycardia circuit still remains challenging in these complex cases.

Activation and entrainment mapping have been used as important tools for identifying the circuits and critical isthmuses involved in MRATs. However, annotating local activation times using bipolar EGM recordings can be misleading, since they often mix NF and FF signals, especially in complex EGMs containing both components. Furthermore, entrainment may cause termination or degeneration of atrial tachycardia (AT) into another AT or AF (21).

In such cases, functional substrate mapping, especially ILAM has proven to be more effective than voltage mapping in localizing critical isthmuses. PF analysis can further refine this process by distinguishing NF and FF signals and highlighting high-frequency regions that correlate with areas of slow conduction and potentially helping to determine the critical isthmus where termination can be achieved with fewer ablation lesions (5, 21).

This has led to the concept of combining PF analysis with other tools, such as ILAM and voltage mapping to enhance mapping, identify critical substrates and guide ablation in MRATs. Yorgun and his colleagues showed that deceleration zones (DZs) on ILAM could be identified with a high accuracy using automated PF algorithm in patients with re-entrant AT, which were co-localized with the critical isthmus. They also demonstrated that a PF display of ≥280 Hz identified the termination site of the AT better than the voltage data highlighting the promising role of peak frequency analysis in functional substrate mapping during sinus rhythm to detect the critical isthmus of MRAT (5).

Petzl and his colleagues also conducted a recent study including 17 patients with MRATs. The critical isthmuses in MRATs were characterized by significantly higher PFs than the rest of the atrium, with termination sites typically exhibiting maximal PF values exceeding 530 Hz (mean: >615 Hz). These cutoff values were similar to the PF threshold of 550 Hz proposed by Sudo et al. in his report of 2 cases with complex AT to delineate critical isthmus regions and PV gaps (4, 21).

Interestingly, there was a trend toward a positive correlation between higher global atrial PFs and termination site PFs in the study conducted by Petzl and his colleagues, which could explain the differences in PF cutoff values between studies suggesting that the optimal cutoff value is patient specific and that the PF range should be dynamically adjusted to identify a localized area of very high PF within low-voltage regions rather than adhering to rigid cutoffs. Instead, PF thresholds may be adjusted dynamically to each patient's frequency distribution to identify a localized region of comparatively high PF within low-voltage substrate (21).

Furthermore, PF sites relevant to critical isthmuses and MRATs termination were lower voltage compared to the global atrial voltage, aligning with the observation that high PF areas relevant to arrhythmia isthmuses are consistently located within low-voltage scar regions (<0.5 mV) (21). While high-frequency signals may also appear in healthy myocardium (e.g., ganglionated plexi), these are usually high-voltage and not linked to arrhythmia circuits (22). Therefore, targeting high-frequency signals specifically within or at the borders of scar areas is most meaningful.

Kawaji and colleagues, in a two-case series using PF analysis with a straight pulsed-field ablation catheter during cavotricuspid isthmus ablation, showed that high peak frequencies in pre-application local potentials are useful indicators of tissue proximity and effective cavotricuspid isthmus ablation. They also suggested that 150 Hz may be an appropriate cutoff value (23).

Finally, integrating PF mapping with established mapping techniques may improve the efficiency of MRAT mapping and ablation by helping to focus lesion delivery on regions most likely to represent the critical isthmus. This approach may be particularly useful during sinus rhythm or when tachycardia induction is unsuccessful and may help avoid unnecessary ablation lesions delivered and the creation of extensive linear lesions across healthy myocardium, which could predispose to gaps and arrhythmia recurrence.

**Figure 2:**
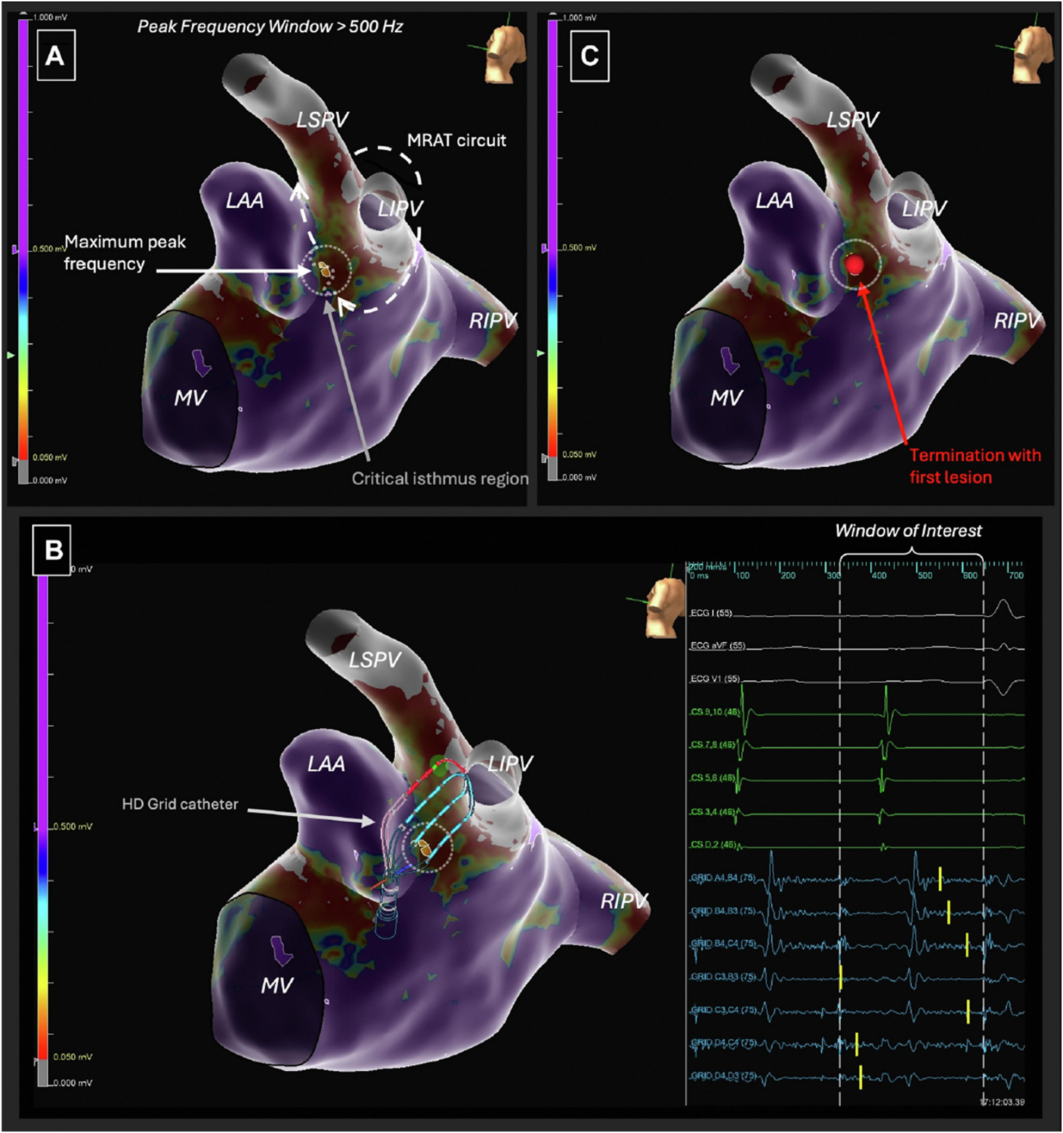
Electro-anatomical voltage map depicting the following: **(A)** case of LA MRAT spinning around the left PVs in between LAA and the veins, in a patient who had undergone previous PVI. PF window adjusted to highlight a confined area of the highest relative frequency within the scar (set in this example to >500 Hz). **(B)** Signals of the HD-Grid catheter depict the complex fractionated and high-frequency signals. Yellow lines on the HD-Grid tracings (blue) indicate the PF component of each signal, to which the OTNF algorithm annotated to create the map. **(C)** Arrhythmia terminated with the first ablation lesion (red dot) targeted precisely at the area of highest frequency. A few additional consolidation lesions were applied subsequently, and the arrhythmia was non-inducible afterward. Reproduced from [Adrian M. Petzl et al.], with direct permission from the author (21).

Figure 2 shows an example of the role of PF in guiding mapping and ablation of a case of LA MRAT spinning around the left PVs in between LAA and PVs, in a patient who had undergone previous PVI (21).

Table 1 summarizes published studies evaluating the role of PF mapping in atrial arrhythmias.

**Table 1:** Current evidence of PF mapping in atrial arrhythmias.

Arrhythmia type	Clinical substrate/target	Reported PF cut-off values (Hz)	Role of PF mapping	Outcome data/diagnostic accuracy
Atrial Fibrillation (AF)	Pulmonary vein (PV) reconnection after PVI	223 (Kuo)	Differentiation of near-field PV signals from far-field LA activity; objective visualisation of PV reconnections gap	PF-guided mapping reduced the number of ablation applications. Higher sensitivity and PPV than voltage mapping for gap detection (Kuo). PV re-isolation was achieved with a single lesion in ∼63% of patients (Okajima)
		240 (Merino)		
		300 (Ting)		
		315–437 (Okajima)		
	Baseline mapping: Atrial vs PV tissue characterization	PV: ∼346	Quantitative distinction between PV and LA electrograms	Demonstrated physiological PF gradients supporting NF/FF discrimination
		LA body: ∼326		
	Low-voltage zone (LVZ) characterisation	≤214 → fixed remodelling	Differentiation of fixed scar vs functional substrate	Improved substrate specificity; reduced overestimation of LVZs compared with voltage mapping alone
		215–236 → functional remodelling		
		≥244 → non-LVZ		
	Left atrial posterior wall isolation (LAPWI)	≥300	Identification of optimal ablation lines and endocardial gaps	Higher LAPWI success with fewer RF applications and reduced procedure time
Macro-re-entrant AT (MRAT)	Critical isthmus localization	≥280 (Yorgun)	Identification of slow-conduction zones and deceleration zones within scar	Arrhythmia termination is frequently achieved at sites of maximal PF, often with the first lesion PF ≥280 Hz identified termination sites more accurately than voltage mapping (Yorgun); quantitative sensitivity/specificity not reported
		530–615 (Petzl)		
		∼550 (Sudo)		
	Functional substrate mapping in sinus rhythm	Patient-specific (relative highest PF within LVZ)	Augments ILAM by distinguishing near-field from far-field signals	Improved identification of the critical isthmus when the tachycardia is non-inducible

### 2.2 Ventricular arrhythmias (VAs)

#### 2.2.1 Idiopathic VAs

The emerging role of catheter ablation as a first-line treatment for a large group of patients with idiopathic VAs is increasing, offering high efficacy and low complication rates (24). Conventional mapping strategies rely on identifying the site of earliest LAT with QS morphology on the unipolar EGM. With the development of PF mapping, an additional advantage has emerged in the ability to analyse EGM sharpness, enabling differentiation between NF and FF signals.

**Figure 3:**
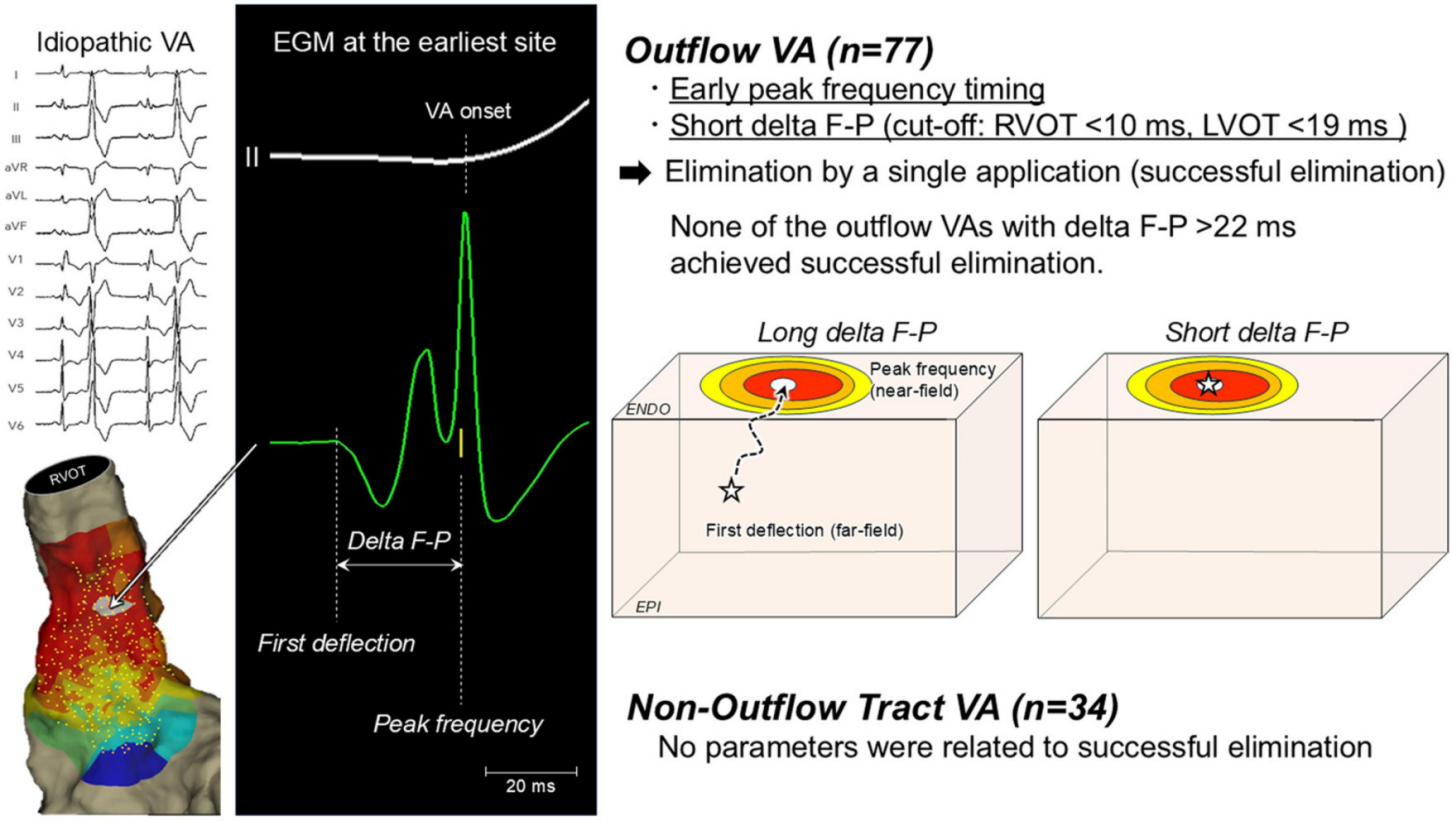
Delta F–P parameter for predicting successful idiopathic outflow tract VA elimination by a single ablation application. Reproduced from [T. Nishimura et al.], under the terms of the Creative Commons Attribution–Non-Commercial License (25).

Recently, Nishimura suggested a new unique parameter; delta between the first deflection and peak frequency (delta F-P) as a predictor of successful endocardial ablation site of outflow tract VAs (Figure 3). Delta F-P can be measured from a single bipolar EGM and is not influenced by the QRS onset which could be very valuable in predicting successful VA elimination when the QRS onset is unclear. Further investigation is warranted to validate this novel parameter especially in those originating from deep intramural foci (25).

#### 2.2.2 Scar related VAs

Identification of critical myocardial isthmus sites for VT maintenance is the primary endpoint during substrate mapping. Besides the interest in functional mapping during sinus rhythm, activation mapping still plays a crucial role especially if the VT is inducible and mappable.

Contemporary substrate mapping techniques in the era of high density mapping using ILAM and decrement-evoked potential (DeEP) mapping have emerged as effective tools for accurately identifying critical isthmuses, particularly given the limitations of entrainment and activation mapping, especially in the setting of haemodynamic instability or lack of inducibility providing greater specificity for identifying arrhythmogenic tissue within myocardial scar and enabling effective VT ablation with more localised ablation target region (26). However, some issues such as signal noise and artifacts may still necessitate manual adjustments of annotation sensitivity or placement which can be time-consuming in the era of multielectrode mapping, where electroanatomic maps often contain thousands of points. Furthermore, it is not always certain whether the latest component of the bipolar EGM truly represents local activation or FF activity.

The use of a PF algorithm based on WT time-frequency analysis in addition to those techniques has emerged as innovative tool to address this issue and accurately annotate fractionated signals by assigning local activation to the EGMs PF and hence reliably differentiating between NF and FF components of complex intracardiac EGMs (1, 3).

Many studies were performed to evaluate the effectiveness of such algorithm, its role in VT mapping and ablation and also defining the cutoff values of the PF via its correlation with the well-known methods such as late potentials, DeEP mapping, mid diastolic isthmus and termination site of VT (2, 3, 27).

Payne et al. demonstrated that within low-voltage regions, markers of VT substrate such as late potentials and local abnormal ventricular activations exhibited higher PF compared to normal tissue or scar without these features. Using this observation, they identified an optimal PF cutoff of >220 Hz during ILAM to detect late potentials and local abnormal ventricular activations with high sensitivity (91%) and specificity (85%). Furthermore, by applying the OT algorithm with this cutoff prospectively in a cohort of 10 VT ablation patients, they found a strong overlap between ILAM-defined DZs and PF-defined zones supporting PF as a reliable marker for VT substrate (2).

**Figure 4:**
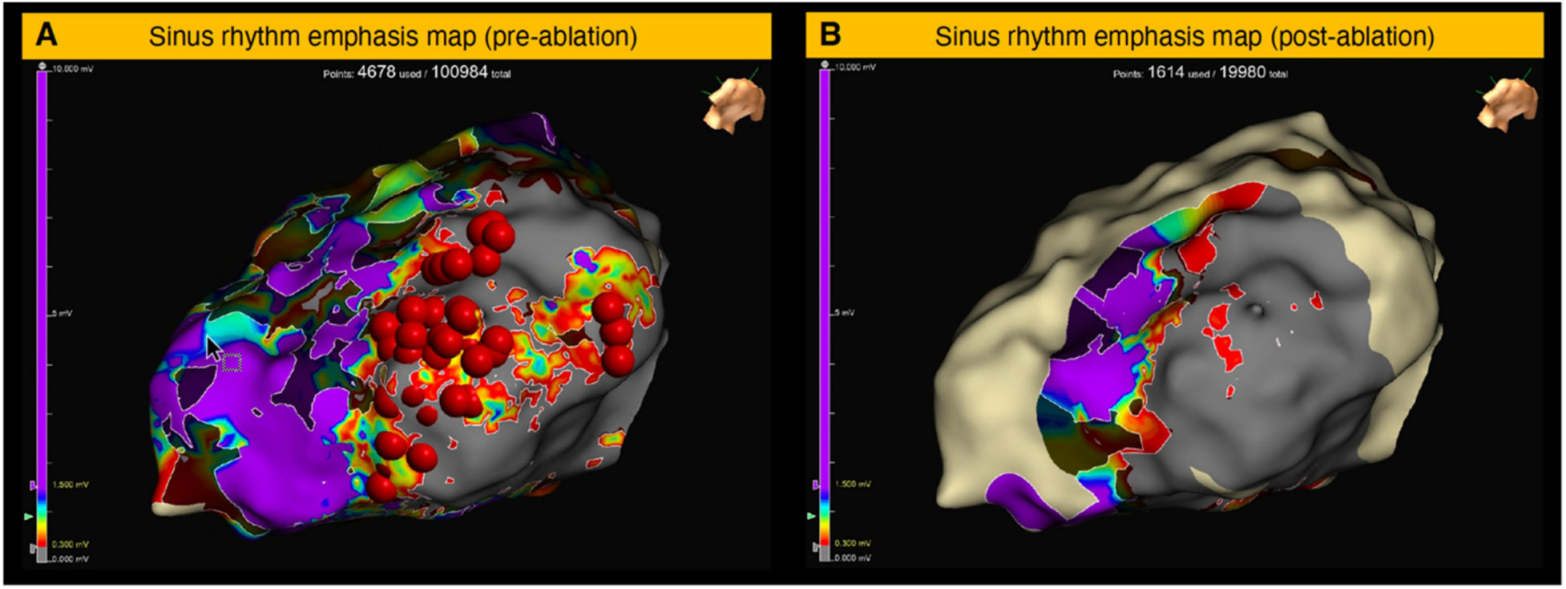
Emphasis maps pre- and post-ablation for a patient with ICM and scar related VT. SR substrate map with PF ≥ 200 Hz was emphasised. All ablation lesions were within 5 mm of the low voltage-high PF region. Post-ablation re-map shows a near complete elimination of low-voltage–high-PF region. Reproduced from [J. Mayer et al.], with direct permission from the author (3).

Similarly, Mayer et al. has shown in multicentre cohort that PF substrate mapping can accurately identifies critical components of the VT circuit but with different threshold of ≥200 Hz (Figure 4) as it was directly correlated to the critical VT isthmus, as opposed to LPs and LAVAs. Furthermore, bespoke offline analysis enabled PF analysis of all map EGMs, reducing the risk of bias through manual verification. Additionally, they proved that the diagnostic performance of substrate-based PF map improves in Amiodarone-naïve compared to amiodarone-treated patients and that the PF cutoff values within the critical isthmus low-voltage–peak frequency values are lower during right ventricular pacing compared to sinus rhythm or during VT (3).

Tonko et al. has applied NF detection algorithm retrospectively to scar-related re-entry VT maps in 18 patients and compared with manually reviewed maps employing corrected first deflection (FDcorr) for VT activation maps and last deflection (LD) for substrate maps. They found that NF detection correctly located critical parts of the circuit in 77.7% of the cases compared with manually reviewed VT maps as reference. In substrate maps, NF detection identified deceleration zones in 88.8% of cases, which overlapped with FDcorr VT isthmus in 72.2% compared with 83.3% overlap of DZ assessed by LD. Additionally, NF detection mapped the complete diastolic wavefront through the isthmus in only 16.6% and identified VT circuits segments partially in 61.1%. Thus, they hypothesized that NF detection may only result in complete VT maps in a two-dimensional planar activating circuit restricted to the mapped surface, whereas incomplete and/or ‘failed’ NF VT mapping could indicate more complex three-dimensional activation patterns that warrant additional assessment (27).

Cauti et al. also demonstrated the clinical utility of PF mapping using OT technology in a cohort of 69 patients with scar-related VT. Elevated PF values (>405 Hz) during VT correlated with rapid VT termination allowing accurate localization of critical circuit components, including DZs (28). Furthermore, this study found that PF analysis can supply data about “zigzag” wavefront activation at various level of the myocardium (depth and width). The highest frequencies can be used as a surrogate for superficial surviving myocyte stigmata (accessible and vulnerable areas inside slow conductive channels). Conversely, mid-range and low frequencies can be reflective of intramural sites requiring longer RF applications for VT termination within the diastolic channel.

Tonko et al. highlighted the potential role of PF mapping in differentiating epicardial fat from scar-related signals. In a patient with arrhythmogenic right ventricular cardiomyopathy and right ventricular outflow tract VT, automated PF analysis (cutoff > 250 Hz) distinguished true low-voltage, high-frequency EGMs in the epicardial right ventricular outflow tract and peri-tricuspid regions from “false” low-voltage, low-frequency signals caused by epicardial fat, as confirmed by semiautomated Computed Tomography-based adipose tissue imaging (Adas3D, Galgo Medical, Barcelona, Spain). Since epicardial fat can mimic scar and impede effective energy delivery, further studies are needed to validate role of PF and the most accurate thresholds for PF to identify epicardial fat (29).

Finally, automated PF analysis is very promising technology complementary to current mapping standards allowing accurate annotation of fractionated signals during VT mapping and identification of VT critical isthmus sites. However, further studies are still required to validate this algorithm in scar-related VT and to determine the most reliable PF cutoff value for predicting VT termination.

### 2.3 Supraventricular tachycardias (SVTs)

#### 2.3.1 Atrioventricular nodal re-entry tachycardia (AVNRT)

The ablation of AVNRT relies on precise identification of the slow pathway while sparing the compact AV node. Conventional strategies based on anatomical landmarks and EGM criteria, though effective, can be time-consuming. PF mapping offers a novel approach by integrating local EGM frequency and LAT which enables delineation of low-frequency, late-activation sites that correspond to the slow pathway. Recent clinical studies have begun to establish the efficacy and safety of PF-guided ablation in AVNRT.

In a case series of 31 patients, 8 underwent NF-PF–guided ablation. NF-PF guidance identified late, low-frequency perinodal signals, with junctional rhythm achieved more rapidly than with conventional EGM-guided ablation. These findings suggest that perinodal automated NF annotation, demonstrating latest activation with frequency attenuation, can serve as a safe alternative slow pathway target for successful ablation in AVNRT (30).

Kawaji et al. also investigated PF characteristics at successful slow pathway ablation sites. In a 10-patient discovery cohort, these sites showed lower peak frequencies than both the His bundle and conventional ablation sites, with findings consistent across AVNRT and sinus rhythm, indicating PF stability. A 5-patient validation cohort using cryoablation confirmed that low-frequency signals (∼199 Hz) marked successful targets and required fewer applications than conventional cryoablation. Overall, the study demonstrates that low-frequency PF signatures reliably identify the slow pathway and are applicable across different ablation energy sources (31).

**Figure 5:**
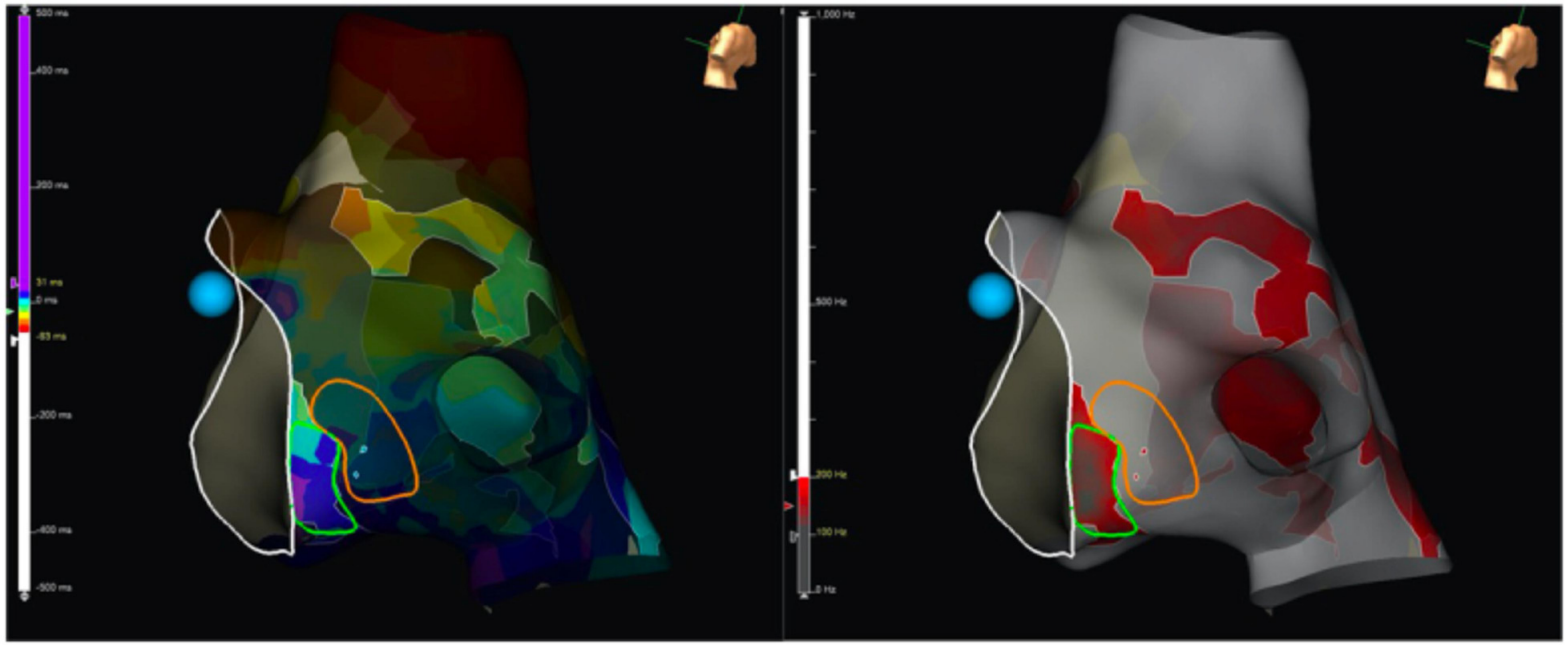
Late activation and PF maps for a patient with typical AVNRT. PF maps were computed online prior ablation and displayed on the activation map with shadowed areas where PF was <200 Hz (figure, left panel). Focal radiofrequency application (was directed successfully to the areas of late activation (left panel) and low frequency (right panel) Reproduced from [A. Handayani et al.], under the terms of the Creative Commons Attribution–Non-Commercial License (32).

In a prospective study of 20 typical AVNRT patients, online PF maps integrated with activation maps revealed discrete late-activation, low-frequency regions in 18 cases. PF values at these regions were significantly lower than in adjacent atrial sites and ablation at these sites successfully induced junctional rhythm, demonstrating that PF mapping reliably identifies effective targets for slow pathway ablation (Figure 5) (32).

Takahashi et al. proposed that high-frequency sites highlighted at the tricuspid annulus (4–5 o’clock position) on PF maps could represent novel target for slow pathway ablation, offering improved safety, efficiency, and success rates especially in challenging cases with narrow Koch's triangle. In a prospective study of 37 AVNRT patients, they compared PF-guided ablation (*n* = 17) with conventional methods (*n* = 20) showing that ablation at these high-frequency sites, located away from the His bundle, resulted in earlier-onset slower junctional rhythm and more frequent elimination of dual AV nodal physiology (33).

Taken together, these studies provide converging evidence that PF mapping can accurately delineate the slow pathway substrate in AVNRT and might be of benefit especially in challenging cases with high risk of atrioventricular block. However randomized controlled studies will be essential to validate these findings and to determine whether PF-guided ablation improves long-term outcomes beyond conventional methods or not.

#### 2.3.2 Accessory pathways

Accessory pathways are a key substrate for SVTs and precise localization is essential for successful ablation, especially in complex regions such as the tricuspid and mitral annuli or coronary sinus. Conventional mapping, based on early activation or Kent bundle potentials, can be limited by small signals or complex conduction.

Recent case reports suggested that PF mapping might aid accessory pathway ablation by delineating high-frequency sites and distinguishing NF from FF signals, potentially improving outcomes in challenging cases.

In one example, a 60-year-old man with recurrent narrow QRS tachycardia underwent open-window mapping (OWM) with an HD Grid™ catheter, which revealed continuous potentials along the tricuspid annulus. Conventional OWM -guided ablation transiently suppressed tachycardia but integrating a PF map (cutoff 350 Hz) identified a slightly more ventricular site that was successfully ablated, permanently eliminating retrograde conduction (34).

Similarly, in a 54-year-old patient with intermittent pre-excitation, PF mapping highlighted Kent bundle potentials at the mitral posterior annulus and coronary sinus. In addition, the peak frequency at the endocardial site was moderate and spread over a wide area with the same frequency on the map, indicating that the origin of the Kent bundle was located far from the endocardium. In contrast, the higher peak frequency and narrower highlighted area in the coronary sinus than in the endocardial area in the LA suggests that the Kent bundle passes through the epicardial side. Initial LA endocardial ablation transiently suppressed the delta wave, but PF-guided ablation within the coronary sinus achieved durable elimination of the accessory pathway without complications, demonstrating the method’s ability to resolve complex conduction patterns that are difficult to identify with conventional mapping alone (35).

Together, these cases highlight the potential of PF mapping using OT to enhance the precision of accessory pathway localization and ablation. By integrating high-frequency analysis with conventional activation and open-window mapping, operators can better identify the true pathway conduction sites, even when anatomical or EGM features are ambiguous. This approach may improve procedural success and reduce recurrence, particularly in anatomically challenging regions or in patients with complex conduction patterns. Table 2 summarizes published studies evaluating the role of PF mapping in SVTs.

**Table 2:** Current evidence of PF mapping in SVTs.

SVT type	Clinical target	Reported PF cut-off values (Hz)	Role of PF mapping	Outcome data/diagnostic accuracy
AVNRT	Slow pathway localisation (low-frequency targets)	∼199 < 200	Identification of late, low-frequency perinodal signals	Faster junctional rhythm onset; fewer RF or Cryo applications; safe slow-pathway ablation
	Alternative slow pathway targets (TA 4–5 o’clock)	Relatively higher PF than the adjacent atrium	Identification of anatomically safer ablation sites distant from the His-bundle	Earlier junctional rhythm; higher elimination of dual AV nodal physiology
Accessory pathways	Tricuspid annulus, mitral annulus, and coronary sinus	∼350 (case-based)	Differentiation of near-field accessory pathway potentials from far-field atrial/ventricular signals	Successful ablation after failure of conventional activation or open-window mapping

## 3 Limitations and gaps in knowledge

The EnSite™ OTNF detection PF algorithm offers a promising potential for a more precise substrate mapping with improvement in the ablation outcomes. However, as with any emerging technique, its clinical application is still evolving, and several limitations must be acknowledged. Addressing these challenges will be essential to define its true role and establishing its value in routine clinical practice.

Signal frequency mapping techniques including PF mapping are inherently sensitive to noise, which may obscure true spectral peaks or introduce spurious ones, particularly in low-amplitude EGMs with a low signal-to-noise ratio. These limitations are not specific to any proprietary algorithm but reflect general challenges of frequency-based signal analysis; therefore, robust filtering strategies, advanced signal-processing approaches, and optimization of the peak-to-sidelobe ratio are required to improve mapping reliability (36).

Adequate catheter–tissue contact is essential for recording clear and reliable EGMs, irrespective of the detection or mapping algorithm used. Suboptimal contact may result in low-frequency or FF signals that obscure true local activity. Omnipolar signal acquisition, which is less dependent on wavefront direction and attenuates continuous low-frequency components, may facilitate improved discrimination of true intramural activity. In addition, features such as PF duplicate detection which prioritizes the point with the highest frequency in the identical region is expected to map more reliably than conventional OT certainty duplicates and may help reduce false signal interpretation; nevertheless, meticulous catheter manipulation remains fundamental for accurate EGM acquisition (28).

In MRAT, PF mapping may help guide operators to critical isthmus sites, though it is best used as an adjunct since high-frequency regions can sometimes lie outside the isthmus. PF should be interpreted alongside voltage maps, with activation mapping and entrainment maneuvers providing confirmation that the identified region is part of the circuit. Variations in cycle length and wavefront direction can alter voltage, conduction velocity, and EGM fractionation, potentially affecting PF annotation and DZ identification (37). Nevertheless, a recent study showed that the spatial distribution of Local ventricular high frequency regions remains consistent despite such variations (38). Currently, the PF tool caps annotations at 716 Hz, although atrial tissue may physiologically exceed this, as observed at several termination sites. Refining the tool to allow higher-frequency annotation could therefore have clinical relevance, pending technical feasibility (5, 21).

In scar-related VT, substrate mapping has traditionally been performed during sinus rhythm or right ventricular pacing, with limited data available for biventricular or LV only activation. The multicenter study PHYSIO-VT demonstrated that the spatial distribution and magnitude of activation slowing are highly dependent on the direction of left ventricular activation. Specifically, activation slowing often corresponds to the site where the wavefront first interacts with infarcted myocardium, and mapping from multiple orthogonal directions can unmask additional areas of activation slowing that may serve as critical isthmus sites. These findings suggest that conventional mapping in sinus rhythm alone may underestimate functionally relevant substrate, and that activation-direction informed strategies increase substrate mapping sensitivity and specificity, with potential implications for improving VT ablation outcomes (39).

The OTNF algorithm analyzes peak-to-peak voltages ≥0.04 mV to minimize low-energy, high-frequency noise, though higher-frequency noise above this threshold may still be included. Importantly, low-frequency NF EGMs may still represent clinically relevant intramural substrate rather than noise alone. Distinguishing clinically relevant intramural signals from low-frequency FF activity remains challenging, and the relationship between frequency content, myocardial depth, and optimal ablation strategy has not been systematically validated. Further studies are required to clarify how NF and PF metrics should be weighted when targeting complex three-dimensional VT circuits.

The potential of PF-guided low-voltage substrate mapping to enhance VT ablation procedural outcomes and RF metrics should be evaluated in a randomized controlled trial. Moreover, integrating the OTNF algorithm with ILAM and DeEP approaches may further optimize substrate mapping performance and offer deeper insights into the intricate 3D structure of the critical isthmus (2).

The potential role of PF guided mapping and ablation in idiopathic VAs, AVNRT and challenging accessory pathways including its integration with different mapping tools such as voltage, LAT, and OWM, still requires validation, particularly to determine whether PF-guided ablation improves long-term outcomes beyond conventional methods.

Finally, most existing studies are retrospective, include small patient cohorts, and report non-randomized outcomes based on workflows from high-volume centers. There is also no consensus on cutoff values for “significant” PF to distinguish NF from FF signals in either chamber during sinus rhythm or tachycardia, leaving current evidence limited. Data on patient-specific characteristics, such as medical history, are also lacking, and the impact of specific maneuvers, such as pacing, on PF signals remains unclear. Large, multicenter trials are needed to determine whether targeting high-frequency areas improves ablation success rates and long-term arrhythmia freedom.

## 4 Conclusion

The PF algorithm represents a promising tool for characterizing arrhythmia substrates by identifying regions of high frequency within re-entrant circuits and other areas of interest. Integrating PF analysis with different mapping technologies including LAT, voltage, ILAM, and DeEP mapping allows for rapid high density mapping, early identification of ablation targets, and reduced ablation time. However, randomized controlled trials are still needed to validate its clinical utility and establish cutoff values for “significant” peak frequency to guide data interpretation and improve the success rate of ablation procedures.
